# Editorial: Key opinions showcase: Gastroenterology

**DOI:** 10.3389/fgstr.2023.1159403

**Published:** 2023-03-01

**Authors:** Colm Antoine O. Morain

**Affiliations:** Faculty of Health Sciences Trinity College Dublin, Dublin, Ireland

**Keywords:** inflammatory bowel disease, functional bowel disease, colorectal cancer screening, Helicobacter pylori, Helicobacter pylori treatment

Gastroenterology is not recognised sufficiently. The reasons are multi factorial including the busy schedule of Gastroenterologists which includes endoscopy and management of a wide range of gastro intestinal conditions. In some jurisdiction’s gastroenterologist take part in a general internal medical rota which leaves little time to lobby.

It is estimated that every citizen will need to visit Gastroenterologist at least once in their lifetime. Up to 30% of all patients presenting to emergency hospital departments have a gastro intestinal cause for their presenting complaint. However the substantial burden of digestive disease has gained little attention in terms of policy, research and funding. The launch of Frontiers in Gastroenterology is welcomed as it will provide a platform to highlight the importance of the speciality. In this showcase edition of which I had the honour to edit, important topics in Gastroenterology are addressed by key opinion leaders.

The European registry on Helicobacter pylori management (Hp-EuReg) monitored indications diagnoses and treatment of H pylori for the past 10 years which has influenced the management of this infection by practicing gastroenterologist worldwide. In their review Nyssen et al. highlight the published relevant findings of their work over the last decade. In this period over 25 studies from the registry have been published. Their publications have contributed to the improvement in H pylori eradication rates and have been the basis of consensus guidelines. The discovery of H Pylori over 40 years has led to a paradigm shift in that eradicating the bacteria cures peptic ulcers and prevents gastric cancer. The treatment has evolved since its discovery and now evolves quadruple therapy. The threshold for acceptance of a chosen treatment to cure infection should have and eradication rate of 90%. There are challenges in achieving this goal including antibiotic resistance and compliance to treatment. The registry contains data from over 50.000 patients from 28 counties. Over 90% eradication rates have been obtained with 10 or 14 day bismuth quadruple therapy or 14 day concomitant treatment. The addition of bismuth to a 14 day standard triple therapy with clarithromycin and amoxicillin eradicates in 90% of patients suggesting that bismuth may overcome antibiotic resistance. Further evaluation suggests levofloxacin as an effective second line treatment. Ideally culture should be performed to guide second line treatment but this is not universally available and was performed in less the 10% of cases in the registry however clarithromycin has risen to over 15% and should be avoided unless sensitivity tests have been performed.

A thought provoking review contributed by Stange discusses the current and future aspects of Inflammatory bowel disease. Stange admits that a major breakthrough on treatment is the introduction of biological therapy however these are only partially successful in inducing long term remission. The immunological hypotheses is that IBD represents an abnormal response to a normal stimulus in a genetic susceptible host. He contends that the focus should be at the mucosal antibacterial barrier which could lead to a cure. The long established 5 amino salicylates, steroids and thiopurines have been eclipsed by investigation of cytokine mediators and the lymphocyte/endothelial adhesion molecules leading anti TNF which not only neutralise circulating TNF but induces apoptosis of inflammatory cells carrying TNF. Later antibodies to inhibit interleukins alpha 4 beta 7 integrin blocking lymphocyte entry into the gut have been partially successful in treatment of IBD. Oral administered drugs blocking the JAK kinase pathway and ozanimod limiting lymphocyte exit from lymph nodes. All of these drugs have limitation and side effects and suffer from loss of response and does not prevent disease progression. This should encourage new therapeutic approaches and accepting that IBD is a barrier disease. The identifications of genes associated with IBD have been helpful but significant progress in the genetic associations has not translated to progress in clinical practice. The environment has a major role increasing and reducing risk of IBD. The microbiome is the target of the immune response however alterations in the microbiome may be secondary rather than primary. His comprehensive review suggests mucins to be involved and favouring mucus enhancing microbes targeting anti-microbial peptides and restoring Paneth cell function which could lead to a cure of inflammatory bowel disease.

Functional bowel disease is common and up to 40% of the population suffer from this condition. It has a major impact on the quality of life of some sufferers and causes a significant burden on health services in terms of cost and management. In the review by Walker et al. they explore the relationship of functional bowel disease with inflammatory bowel disease (IBD) as they can co-exist and there is an overlap of symptoms. The cause of functional bowel disease in IBD is multi factorial. it is important to recognise it and to distinguish from a flare up of IBD. This may prevent escalation of therapy that is expensive and not without risk. This differential diagnosis can be helped non-invasive tests such as C reactive protein and stool calprotectin. The gut brain axis gastro intestinal motility microbiome dysbiosis previous bowel resection impaired intestinal permeability immune mediated system activity and visceral hyper sensitive have all been implicated in their cause. Genetic factors have been suggested as there is a higher incidence of IBS amongst monozygotic twins than dizygotic twins but having a parent with IBS is a stronger risk factor. Management of IBS includes lifestyle changes diet, exercise and psychological support and from a pharmacological intervention to control symptoms.

In a subject in which gastroenterologist have contributed significantly is screening for colorectal cancer (CRC). Population based screening is based on a 2 step process of offering an faecal immune chemical test (FIT) to individuals between the ages of 50 and 74 and if the test is positive a colonoscopy is performed. In their review Kortlevor et al. discuss the risk models for CRC screening and the obstacles to enable wide spread implementation in existing CRC screening programmes. They suggest a test more sensitive then FIT alone and acceptable cost effective by applying risk factors to a defying population. Risk based screening could be applied at entry or in follow up utilising risk-based surveys. Risk factor are age, BMI, family history, alcohol consumption, smoking habits, diets and medications and if present these patients may require a colonoscopy at a lower fit cut of level. However successful screening programmes depends on participation rates filling in a questionnaire may reduce this rate. These patients at risk if identified may require shorter intervals for colonoscopy longitudinally. Other faecal markers multi target stool DNA calprotectin and microbiome analysis are been evaluated. Markers from peripheral blood include messenger RNA micro RNA cell free DNA are being assessed.

## Author contributions

The author confirms being the sole contributor of this work and has approved it for publication.

